# Supplementary material for: Overcoming Pain and Kinesiophobia: Unlocking the Path to Better Knee Osteoarthritis Rehabilitation
Source: Biomedicines. 2025 Jun 1;13(6):1361. doi: 10.3390/biomedicines13061361 (PMC12189791; doi:10.3390/biomedicines13061361)
Supplement: Supplementary file 1 [file biomedicines-13-01361-s001.zip › biomedicines-3538320-supplementary.pdf]

Supplementary table S1. Regression coefficients, covariances and variances of model M1 between VAS and TSK scores or between VAS and KOOS scores.

predictor		outcome	β	β'	SE	*z*	*p(z)*
**regressions**							
VAS-T0	→	VAS-T1	0.640	0.521	0.148	4.325	<.001
VAS-T0	→	TSK-T1	1.144	0.136	0.997	1.148	0.251
TSK-T0	→	VAS-T1	0.047	0.316	0.018	2.624	0.009
TSK-T0	→	TSK-T1	0.665	0.645	0.122	5.459	<.001
VAS-T1	→	VAS-T2	0.721	0.781	0.117	6.161	<.001
VAS-T1	→	TSK-T2	1.936	0.243	0.948	2.041	0.041
TSK-T1	→	VAS-T2	-0.012	-0.092	0.017	-0.728	0.466
TSK-T1	→	TSK-T2	0.712	0.615	0.138	5.168	<.001
**covariances**							
VAS-T0	↔	TSK-T0	5.427	0.254	3.483	1.558	0.119
VAS-T1	↔	TSK-T1	4.453	0.309	2.382	1.869	0.062
VAS-T2	↔	TSK-T2	2.572	0.207	2.007	1.281	0.200
**variances**							
VAS-T0			2.610	1.000	0.584	4.469	<.001
VAS-T1			2.136	0.544	0.478	4.469	<.001
VAS-T2			1.535	0.458	0.343	4.475	<.001
TSK-T0			174.660	1.000	39.055	4.472	<.001
TSK-T1			97.011	0.522	21.692	4.472	<.001
TSK-T2			100.690	0.403	22.515	4.472	<.001

Model fit

χ^2	df	$p(\chi^2)$	AIC	BIC
4.653	4	0.325	1378.5	1417.3

predictor		outcome	β	β'	SE	*z*	*p(z)*
**regressions**							
VAS-T0	→	VAS-T1	0.650	0.530	0.163	3.975	<.001
VAS-T0	→	KOOS-T1	-2.179	-0.237	1.063	-2.050	0.040
KOOS-T0	→	VAS-T1	-0.030	-0.189	0.021	-1.419	0.156
KOOS-T0	→	KOOS-T1	0.736	0.612	0.139	5.287	<.001
VAS-T1	→	VAS-T2	0.789	0.855	0.133	5.955	<.001
VAS-T1	→	KOOS-T2	-1.264	-0.213	0.636	-1.987	0.047
KOOS-T1	→	VAS-T2	0.022	0.182	0.018	1.266	0.205
KOOS-T1	→	KOOS-T2	0.562	0.708	0.085	6.611	<.001
**covariances**							
VAS-T0	↔	KOOS-T0	-7.602	-0.381	3.373	-2.254	0.024
VAS-T1	↔	KOOS-T1	-8.648	-0.558	2.806	-3.082	0.002
VAS-T2	↔	KOOS-T2	-3.429	-0.478	1.258	-2.727	0.006
**variances**							
VAS-T0			2.610	1.000	0.584	4.469	<.001
VAS-T1			2.383	0.607	0.533	4.471	<.001
VAS-T2			1.495	0.447	0.334	4.476	<.001
KOOS-T0			152.228	1.000	34.039	4.472	<.001
KOOS-T1			100.780	0.458	22.535	4.472	<.001
KOOS-T2			34.453	0.249	7.704	4.472	<.001

Model fit

χ^2	df	$p(\chi^2)$	AIC	BIC
6.436	4	0.169	1311.8	1350.7

Note: β = regression coefficient for raw data; β’ = regression coefficient for standardized data; SE = standard error; AIC = Akaike information criterion; BIC = Bayesian information criterion; VAS = visual analog scale; TSK = Tampa scale of kinesiophobia; KOOS = knee injury and osteoarthritis outcome scale; T0 = before treatment; T1 = after treatment; T2 = follow-up.
